# Sex-Based Differences in the Presentation of Myocardial Infarction

**DOI:** 10.7759/cureus.16906

**Published:** 2021-08-05

**Authors:** Jeremy Mayfield, Muneet Gill, Helen J Zhang, Latha Ganti

**Affiliations:** 1 Emergency Medicine, HCA Healthcare Graduate Medical Education Consortium Emergency Medicine Residency Program of Greater Orlando, Orlando, USA; 2 Emergency Medicine, Osceola Regional Medical Center, Kissimmee, USA; 3 Emergency Medicine, Brown University, Providence, USA; 4 Emergency Medicine, Envision Physician Services, Plantation, USA; 5 Emergency Medicine, University of Central Florida College of Medicine, Orlando, USA

**Keywords:** non st-segment elevation myocardial infarction, sex-based differnces in coronary disease, sex-based differences in acute coronary syndrome, chest pain, emergency cardiology

## Abstract

The authors report a case of a middle-aged female presenting with a chief complaint of shoulder pain. Workup revealed a non-ST-segment elevation myocardial infarction. The typical symptoms of myocardial infarction and the evaluation of a non-ST-segment elevation myocardial infarction are discussed. The authors highlight the sex-based differences in the presentation of myocardial infarction and remind us to keep a broad differential and consider atypical presentations.

## Introduction

Myocardial infarction is defined as a decrease in or absence of blood flow to any area of the heart, resulting in damage to the muscle. This occurs because the formation of plaques in the interior walls of the arteries reduces blood flow to the heart, depriving it of oxygen [1]. Myocardial infarction classically presents as a deep, visceral chest pain, which is typically described as heavy, squeezing, tightness, crushing, and sometimes stabbing or burning pain. The pain may radiate to corresponding dermatomes such as the epigastrium, shoulders, arms, back (interscapular region), lower jaw, and neck [2]. Some of the feared complications of myocardial infarction include mechanical, arrhythmic, ischemic, and inflammatory (early pericarditis and post-myocardial infarction syndrome) sequelae, as well as left ventricular mural thrombus [3]. Moreover, approximately 5% of patients with myocardial infarction will suffer cardiogenic shock, which has a mortality of ≥30% [4]. Thus, a timely diagnosis is imperative.

The typical presentation discussed above of myocardial infarction is often not present in women, as this case highlights. Thus, it is important to take this discrepancy into consideration with female patients. The authors discuss their patients in the context of sex-based differences in presentation for myocardial infarction.

## Case presentation

A 63-year-old female with a 60-pack-year smoking history presented to the emergency department with left shoulder pain. The shoulder pain started two weeks prior after she was lifting a box. The pain was located on the anterior portion of her left shoulder. The area was exquisitely tender to palpitation, and she had limited range of motion of her left shoulder due to the pain. The patient also endorsed one episode of vomiting that occurred one day prior when the pain was at its maximal intensity 10/10 and spread to her left chest area. She rated her pain a 5/10 at rest. She described the pain as tight. On assessment, the patient was sitting comfortably in the bed. Vital signs were: blood pressure in the right arm 146/98 mmHg, left arm 144/96 mmHg, heart rate 117 beats per minute, pulse oximetry 100% on room air, respiratory rate 18 breaths per minute, and temperature 36.9^0^C. Laboratory analysis was unremarkable (Table 1).

**Table 1:** Patient's initial laboratory results

	Reference Ranges	Laboratory Analysis
Sodium (mmol/L)	136-145	132
Potassium (mmol/L)	3.7-5.1	3.9
Chloride (mmol/L)	98-107	105
Carbon dioxide (mmol/L)	21-32	21
Blood Urea Nitrogen (mg/dL)	7-18	10
Creatinine (mg/dL)	0.55-1.3	0.78
Glucose (mg/dL)	74-106	325
Calcium (mg/dL)	8.4-19.1	8.9
Troponin I (ng/mL)	Equal/less than 0.05	4.82
White Blood Cell count (10^3^/µL)	4.0-10.5	22.7
Red Blood Cell count (10^6^/µL)	3.93-5.22	4.59
Hemoglobin (g/dL)	13.7-17.5	14
Hematocrit (%)	34.1-44.9	43.4
Platelet Count (10^3^/µL)	150-450	276
Immature Gran (%)	0.0-0.4	0.5
Neutrophils (%)	34.0-71.1	89.4
Lymphocytes (%)	19.3-51.7	7.6
Monocytes (%)	4.7-12.5	2.4
Eosinophils (%)	0.7-5.8	0
Basophils (%)	0.1-1.2	0.1

**Figure 1:**
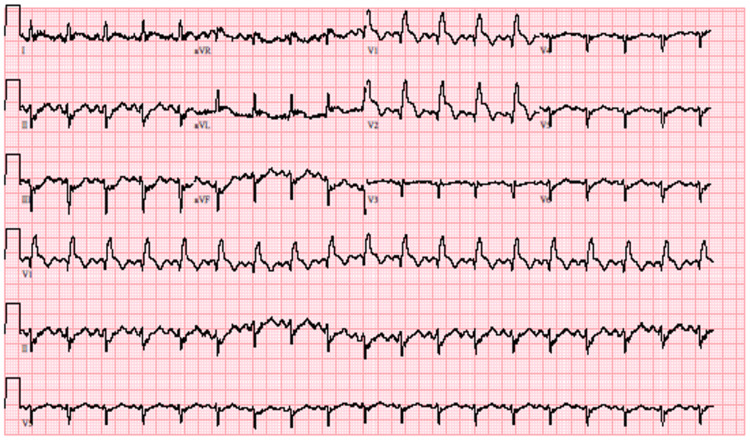
Patient's electrocardiogram demonstrating ST elevation

The electrocardiogram however demonstrated ST elevation in V1-V2 and ST depression in II, III, and aVF (Figure 1).

The patient had never had symptoms like this before. She denied drug use including cocaine or marijuana. She was otherwise healthy, denied fever, chills, cough, diarrhea, hematuria, dysuria, or recent travels. Her only allergy was to fluoroquinolones.

The patient's symptoms were not typical for other emergent causes of shoulder or chest pain such as aortic dissection, pericarditis, pneumonia, pneumothorax, Boerhaave’s syndrome, pneumomediastinum, or shingles. The patient was emergently taken to cardiac catheterization where she was found to have triple vessel disease. Cardiothoracic surgery was consulted, and the patient underwent emergent coronary artery bypass grafting (CABG) due to continued chest pain. Nine days later the patient was discharged home.

## Discussion

**Figure 2:**
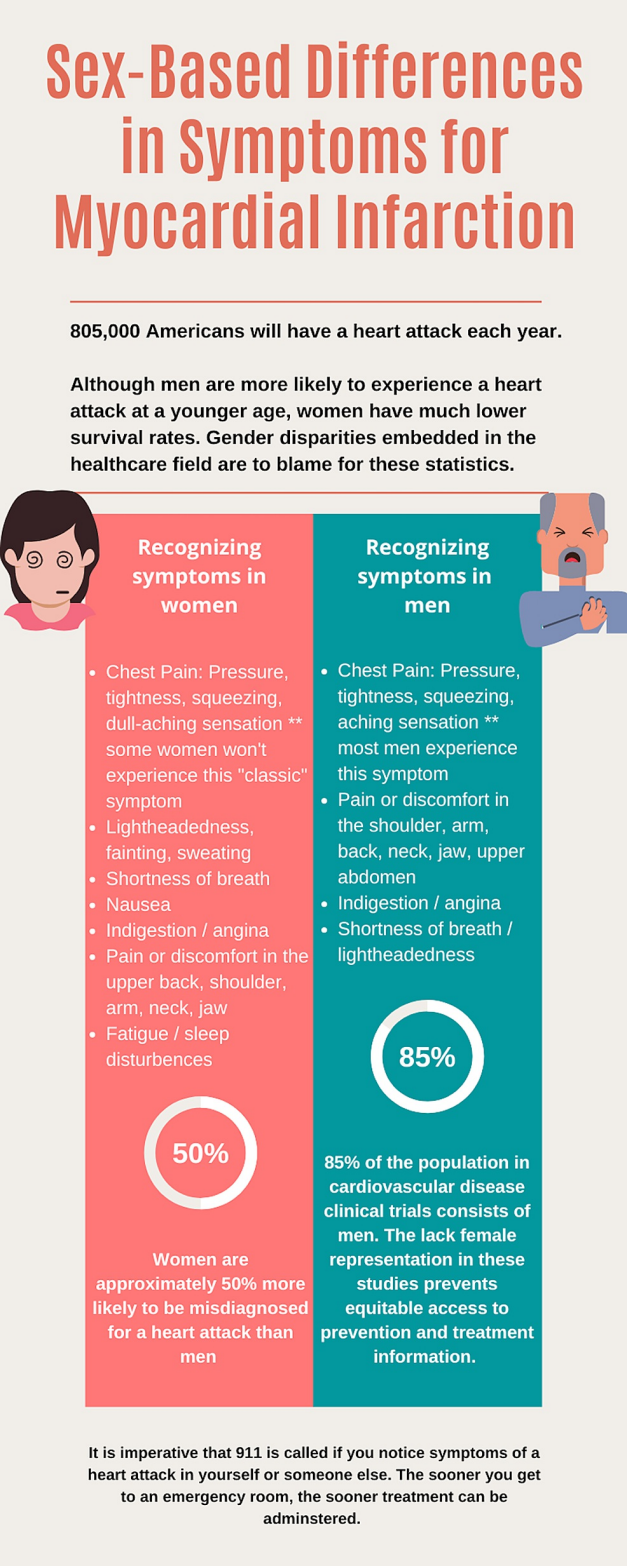
Sex-based differences in symptom presentation for myocardial infarction

This case demonstrates an unusual presentation of myocardial infarction and illustrates the importance of sex-based criteria when it comes to symptoms and diagnosis. Commonly recognized signs and symptoms of myocardial infarction include chest pain, heart palpitations, and radiating arm pain [1]. However, women are far less likely to have chest pain as the primary symptom and instead can present with malaise, nausea, and extremity discomfort [5]. A separate meta-analysis also demonstrated that women are more likely to present with shoulder pain, emesis, and shortness of breath, two of which were observed in our patient [6]. Sex-based differences also exist in electrocardiography, with women presenting with less ST-segment elevation in their electrocardiograms (Figure 2) [7].

More importantly, women also have a higher rate of missed diagnoses; in a study of 563 individuals, approximately 30% of myocardial infarctions were undiagnosed in women compared to 16% in men [8]. This lack of recognition was mainly attributed to less specific cardiac symptoms that were self-reported by the patients. Unrecognized myocardial infarction patients also had higher glucose levels, hypertension, and positive smoking status, which are now used as increased predictors of myocardial infarction in women compared to men. This previous misclassification questions the accuracy of past literature reviews, awareness campaigns, and treatment courses that are typically used in the ED.

In recent years, there has been increasing pressure to characterize the sex-based differences for the initial presentation of myocardial infarction. For example, the description of chest pain is vastly different for men and women. Women more often define their pain as “tight” or a “dull ache,” whereas men describe their pain as “stabbing” [5]. Women also often describe a cluster of symptoms compared to a “classic angina,” meaning that their symptoms may often be mistaken for other illnesses because of a varied presentation [9]. Furthermore, smoking and diabetes are more sensitive risk factors of about two-fold in women compared to men.

Troponin assays are also extremely important in diagnosing myocardial injury and infarction. High sensitivity cardiac troponin assays can more intricately assess variation between sexes, with women having a significantly lower end limit of 16ng/L compared to 34ng/L in men in order to establish a decision threshold [7].

## Conclusions

It has been shown that defining sex-specific criteria is highly useful in distinguishing myocardial infarction from other potential diagnoses. This case illustrates these differences as seen in the patient’s primary symptom of shoulder pain, clustering symptoms of emesis and nausea, and the description of the chest pain. Given the significant differences in symptomatology and lab testing between sexes, it is important for clinicians to consider sex and gender disparities for more efficient diagnosis and treatment.
